# Quality Marker Discovery and Quality Evaluation of *Eucommia ulmoides* Pollen Using UPLC-QTOF-MS Combined with a DPPH-HPLC Antioxidant Activity Screening Method

**DOI:** 10.3390/molecules28135288

**Published:** 2023-07-07

**Authors:** Fengqian Guo, Yichun Yang, Yu Duan, Chun Li, Huimin Gao, Hongyu Liu, Qiping Cui, Zhongyuan Guo, Xiaoqian Liu, Zhimin Wang

**Affiliations:** Institute of Chinese Materia Medica, China Academy of Chinese Medical Sciences, Beijing 100700, China; fengqianguo@126.com (F.G.); yyc199510022023@163.com (Y.Y.); yduan666@163.com (Y.D.); cli@icmm.ac.cn (C.L.); huimin_gao@126.com (H.G.); liuhongyucacms@163.com (H.L.); qipingcc@163.com (Q.C.); 15286838790@163.com (Z.G.)

**Keywords:** *Eucommia ulmoides* pollen, chemical composition, antioxidants, activity determination, quality evaluation, molecular docking

## Abstract

Pollen, as an important component of *Eucommia ulmoides* (EUP), is rich in nutrients and is receiving increasing attention. At present, there are no reports on research related to the chemical composition and quality standards of EUP, and there are significant quality differences and counterfeit phenomena in the market. This study used a UPLC-QTOF-MS system to identify 49 chemical components in EUP for the first time. In the second step, 2,2-diphenyl-1-picrylhydrazyl (DPPH)-HPLC antioxidant activity screening technology was used to identify the main active components of EUP, quercetin-3-*O*-sophoroside (QSH), quercetin-3-*O*-sambubioside (QSB), and quercetin 3-*O*-neohesperidoside (QNH), and their purification, preparation, and structure identification were carried out. Third, molecular docking was used to predict the activity of these components. Fourth, the intracellular ROS generation model of RAW264.7 induced by H_2_O_2_ was used to verify and evaluate the activity of candidate active ingredients to determine their feasibility as Q-markers. Finally, a quality control method for EUP was constructed using the three selected components as Q-markers. The identification of chemical components and the discovery, prediction, and confirmation of characteristic Q-markers in EUP provide important references for better research on EUP and the effective evaluation and control of its quality. This approach provides a new model for the quality control of novel foods or dietary supplements.

## 1. Introduction

*Eucommia ulmoides* Oliver (EU), also known as Du-Zhong in China and Tuchong in Japan, is a traditional medicinal plant that originated in China and is widely distributed in central and southwest China, including Henan, Hunan, Jiangxi, and Shanxi provinces [1]. The bark of *E. ulmoides* (EUB) has been used in traditional Chinese medicine for more than 2000 years. It possesses the pharmacological effects of nourishing the liver and kidney, strengthening the muscles and bones, and preventing miscarriage [2]. The favorable antioxidant activities of *E. ulmoides* have been demonstrated in biological in vivo experiments, with validity against oxidative stress in gastric mucosal injury, chronic hepatotoxicity, diabetes complications, lead-induction, obesity, I/R induced renal and hepatic toxicity, etc., demonstrated [3,4,5,6,7]. In addition to its medical benefits, EU has a high value in developing commercial products. Male flowers of EU (EUF) and EU seed oil have been approved as novel raw food materials by the National Health Commission (NHC) of China. At present, *Eucommia ulmoides* flower natural health-care tea [8] and *Eucommia ulmoides* leaf (EUL) vinegar [9] are common in the market.

The bark, leaves, stems, fruit, and flowers of EU possess an extensive range of pharmacological effects, such as anti-inflammatory [10,11,12], neuroprotective [13,14], and anti-hyperlipidemic [15,16]; treating secondary hypertension [17,18,19]; immunomodulatory effects [20]; and anti-hyperglycemic activities [21].

Natural products have attracted considerable attention as significant resources for preventing oxidative stress-related diseases. Pollen has been recognized as an excellent functional food and feed ingredient [22,23,24] as well as a good source of different bioactive compounds [25,26,27]. The dominant presence and high content of protein, trace elements, minerals, and active ingredients in pollen, as a part of EUF, highlight it as an ideal natural supplement [28]. Previous animal experiments have shown that EUP has antioxidant, antihypertensive, and lipid-lowering effects [29,30]. It is considered to have high development value and broad application prospects in medicine and the healthcare industry. With the increasing attention paid to EUP, the demand for EUP in the market is also gradually expanding. According to research, there are uneven levels of pollen quality and counterfeit products on the market. There have been no reports on its chemical composition and quality control so far. It is necessary to establish appropriate methods to control its quality.

Pollen has significant antioxidant effects and can prevent the occurrence of related diseases by inhibiting the oxidation process [27,31,32]. This study aimed to establish a quality evaluation method for EUP based on antioxidant activity. First, based on an analysis of the chemical characteristics of different parts of EU using (UPLC)–electrospray ionization (ESI) tandem mass spectrometry (QTOF/MS), this paper searched for the characteristic components of EUP. Further, based on DPPH high-performance liquid chromatography (HPLC) antioxidant activity measurement technology, the main active components in EUP were rapidly screened, and the target compounds were separated and prepared. The activity of these components was predicted using molecular docking. Then, their antioxidant activity was verified based on the oxidative damage induced by H_2_O_2_ in vitro to confirm their qualification as Q-markers. Finally, these Q-markers were used to establish a rapid and effective quality control method that can evaluate the quality of EUP and its related health products.

## 2. Results

### 2.1. UPLC-ESI-TOF/MS Analysis

**Figure 1:**
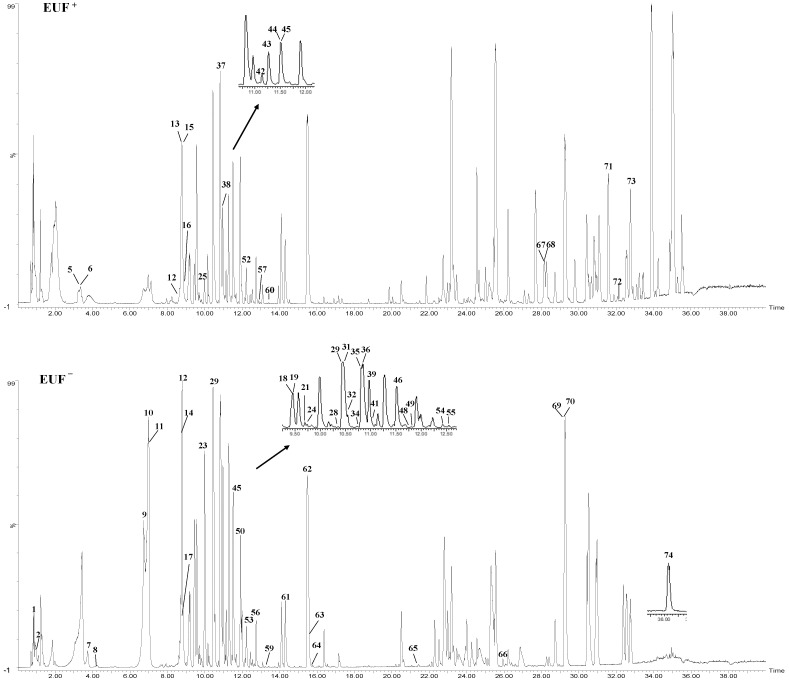
UPLC/QTOF-MS based peak ion diagram (BPI) of different parts of EU. A, EUB; B, EUL; C, EUF; D, EUP; +, positive; −, negative.

In this research, the chemical components in different parts of EU were investigated using UPLC-QTOF-MS/MS, and peak identification was performed. A total of 74 compounds (as shown in Figure 1 and Figure S1 and Table 1), including 21 lignins, 17 iridoids, 17 phenylpropanoids, 13 flavonoids, and 6 other components, were identified or tentatively assigned using UNIFY 1.7 software (Waters Corporation, Milford, CT, USA) by the matching of empirical molecular formulae, quasi-molecular ions, and fragment ions or comparing their characteristic high-resolution mass data with the data from previous publications [33,34,35,36]. The mass error for the molecular ions of all identified compounds was within ± 10 ppm and based peak ion (BPI) diagrams in the negative and positive ion modes are displayed (Figure 1 and Figure S1). The distribution of the compounds was as follows: 62 compounds in male flowers; 49 compounds in pollen; 48 compounds in bark; and 60 compounds in leaves. EUP had 3 lignans, 14 phenylpropanoids, 13 cyclic ether terpenes, 13 flavonoids, and 6 other compounds.

#### 2.1.1. Identification of Lignins

A total of 21 lignins were identified in EUB, EUL, EUF, and EUP. Lignins were the most numerous components in the identified small molecules. When lignins were bombarded with energy, characteristic fragment ions were produced by the loss of a series of glycosyl groups and the internal cleavage of the lignins. For example, (+)-pinoresinol di-*O*-β-d-glucopyranoside exhibited [M + HCOO]^−^ ions at *m*/*z* 727.2450 in the negative mode. The fragment ions of [M – H − Glc]^−^ and [M − H − Glc − Glc]^−^ were detected at *m*/*z* 519.19 and *m*/*z* 357.13. The fragment ion at *m*/*z* 342.1103 and *m*/*z* 151.04 was obtained by internal lignin cleavage.

#### 2.1.2. Identification of Iridoids

Iridoids are distributed in various parts of EU, and 17 iridoids were identified in the positive and negative ion modes. Neutral fragments, such as glucose, glucose residues, H_2_O, CO_2_, CH_3_OH, and CH_3_COOH, represent fragments that are commonly cleaved from the central iridoid core and form [M − H − Glc]^−^, [M − H − Glc]^−^, [M − H − Glc − H_2_O]^−^, [M − H − Glc − O_2_]^−^, [M − H − Glc − H_2_O − CO_2_]^−^, and other ions. For example, the mass spectrometry cleavage products of geniposidic acid yielded the quasi-molecular ion peak at *m*/*z* 373.1132. Then, losses of CO_2_, H_2_O, Glc, etc., formed fragment peaks at *m*/*z* 211.06, 193.05, 167.08, and 149.06. The formation of fragment ions at *m*/*z* 123.04 originated from the rearrangement of γ-H after the rearrangement of the parent ring.

#### 2.1.3. Identification of Phenylpropanoids

A total of 17 phenylpropanoids were identified in EUB, EUL, EUF, and EUP, including caffeic acid, chlorogenic acid, isochlorogenic acid A, protocatechuic acid, syringin, etc. Using caffeic acid as an example, the quasi-molecular ion peak at *m*/*z* 179.0344 continuously lost CO_2_ and H_2_O to form peaks at *m*/*z* 135.04 and *m*/*z* 117.03.

#### 2.1.4. Identification of Flavonoids

A total of 13 flavonoids were identified in different parts of EU. Rutin is the combination of rutinose and the glycoside of quercetin. Its mass spectrometry cleavage products were represented by a quasi-molecular ion peak at *m*/*z* 609.1461 within the primary mass spectrum. The fragment ions of [M − H − Glc]^−^ were detected at *m*/*z* 301.03. The aglycon fragment was a retro-Diels–Alder (RDA) fragment that generated a fragment at *m*/*z* 151.00. In addition, the *m*/*z* 301.03 fragment lost one molecule of H_2_O and CO to generate a fragment ion at *m*/*z* 255.03. The easy-to-lose neutral molecule CO produced a fragment ion at *m*/*z* 227.03.

### 2.2. HPLC-DPPH Analysis

**Figure 2:**
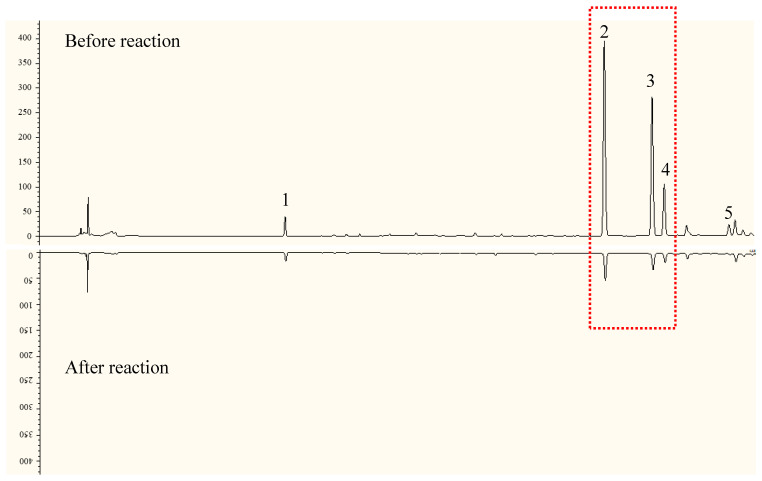
Antioxidant components of 50% methanol extract from EUP by HPLC-DPPH. (the number corresponds to Table 2; Red square, three compounds with the highest response value changes).

**Figure 3:**
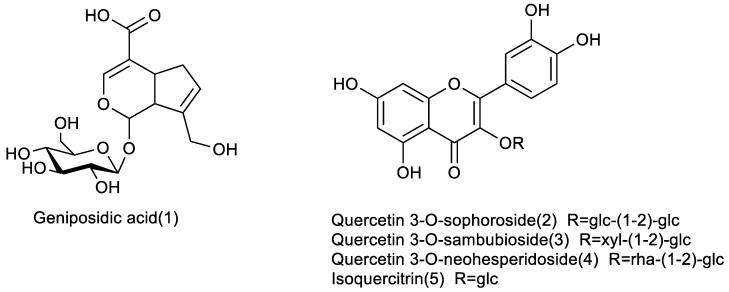
The structure of compounds **1**–**5**.

The chromatogram of the 50% methanol extraction of EUP spiking with DPPH at 254 nm showed that five compounds, **1**–**5**, in the 50% methanol extraction of EUP possessed antioxidant activity (see Table 2, Figure 2 and Figure 3). Compounds **2**–**4** had larger UV absorption at 254 nm before the DPPH reaction, which significantly decreased or even disappeared after the reaction, indicating that these three compounds showed higher antioxidant capacities compared to other components. The ESI-MS results indicate that compounds **2**–**5** had phenolic hydroxyl structures, which were considered to be the main reason for their DPPH radical-scavenging ability.

### 2.3. Preparation and Identification of the Target Antioxidants

Based on the results of the UPLC-Q-TOF-MS and DPPH-HPLC antioxidant testing, three components with significant antioxidant activity and relatively high contents were selected as Q-markers.

#### 2.3.1. Preparation of Q-Markers by Semi-Prep-HPLC

In order to improve the peak shape and resolution, this study investigated the effects of adding different concentrations of acid (0.2% phosphoric acid and 0.4% phosphoric acid) in the aqueous phase. The results showed that a good separation effect could be acquired with 0.2% phosphoric acid. Then, elution solvents (5%, 10%, and 12% acetonitrile–0.2% phosphoric acid (*v*/*v*)) and injection volumes (50 μL, 100 μL, and 200 μL) were investigated to improve the resolution and shorten the separation preparation time in the gradient elution mode. Based on the results, 12% acetonitrile–0.2% phosphoric acid (*v*/*v*) was selected as the best ratio, and 50 μL was the best injection volume. Under the optimized separation conditions, the retention times of the Q-markers in the 50% methanol extract of EUP were 28.64, 34.52, and 36.37 min, respectively.

#### 2.3.2. Structural Identification

**Figure 4:**
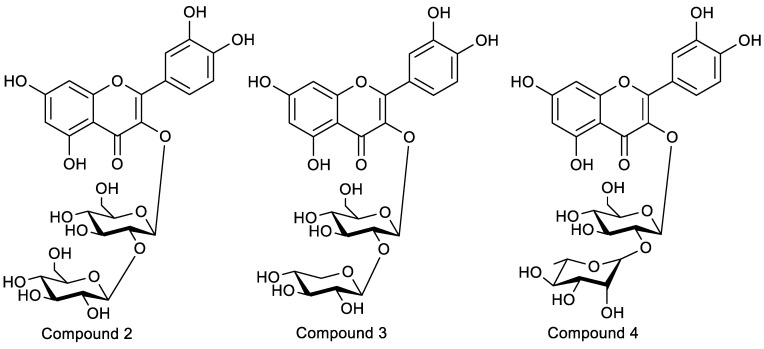
The structure of the 3 Q-markers. Compound **2**: QSH; compound **3**: QSB; compound **4**: QNH.

As Table 2 shown, compound **2** loses a glucose residue to form a fragment ion peak 465 [M + H − Glc]^+^ and continues to lose a glucose residue to form a fragment ion peak 303 [M + H − Glc]^+^ in the positive ion mode. As shown in Table 2, compound **2** loses a glucose residue to form fragment ion peak 465 [M + H − Glc]^+^ and continues to lose a glucose residue to form fragment ion peak 303 [M + H − Glc]^+^ in the positive ion mode. Compound **2** could be tentatively identified as quercetin-3-*O*-sophoroside (QSH) by comparing their chromatographic characteristics, absorption spectra, and previous articles. The monosaccharides of the samples were identified as d-xylose and d-glucose in compound **3** and as l-rhamnose and d-glucose in compound **4** by comparing their retention times with those of the monosaccharide standards. Combined with the results of the mass spectrometry, quercetin di-glycoside (compound **3**) was quercetin-d-xylosyl-d-glucoside, and compound **4** was quercetin-l-rhamnosyl-d-glucoside. The structures of the three Q-markers were confirmed via the NMR of purified flavonoids (Table 3, Figure 4 and Figures S3–S6).

**Compound 2**: yellow powder. ESI-MS (*m*/*z*): 627.1585 [M + H]^+^ (positive), 625.1428 [M − H]^−^ (negative), C_27_H_30_O_17_ (Cal: 626.1483). Compound **2** was identified as 5,7,3′,4′-tetrahydroxyflavone, known as quercetin-3-*O*-sophoroside, by an analysis of the ^1^H-^1^H correlation spectroscopy (COSY), heteronuclear multiple-quantum correlation (HMQC), and heteronuclear multiple-bond correlation (HMBC) spectra (Figures S3 and S4). A large coupling constant (*J* = 7.3 Hz, 7.9 Hz) for the anomeric proton (*δ*_H_ 5.70, *δ*_H_ 4.60) of the glucose in the ^1^H-NMR spectrum suggested a β-configuration in glucose. In the HMBC spectrum, *δ*_H_ 5.70 (H-1 of 3-*O*-Glc) correlated with *δ*_C_ 133.4 (C-3), *δ*_C_ 76.97(C-3″), and *δ*_C_ 98.46(C-1″), and *δ*_H_ 4.60 (H-1 of Glc) correlated with *δ*_C_ 83.14(C-2″) and *δ*_C_ 74.82(C-3‴). The glucose C-2″ signal appeared at *δ*_C_ 83.14, while that of C-2‴ appeared at *δ*_C_ 74.82, suggesting that the inter glycosidic linkage was glucose-(1→2)-glucose. The obtained NMR data are consistent with those of previous research [37,38]. QSH was isolated from EUP for the first time.

**Compound 3**: light yellow powder. ESI-MS (*m*/*z*): 597.1471 [M + H]^+^ (positive), 595.1340 [M − H]^−^ (negative), C_26_H_28_O_16_ (Cal: 596.1378). Compound **3** was also identified as 5,7,3′,4’-tetrahydroxyflavone, known as quercetin-3-*O*-sambubioside, by comparison with previously reported spectral data [39]. The β-configuration of the glucopyranosyl group was indicated based on the large coupling constants (*J*_1,3_ = 7.7 Hz) of the anomeric protons. In the HMBC spectrum (Figures S2 and S4), the correlation of *δ*_H_ 5.68 (H-1 of 3-*O*-glc) with *δ*_C_ 133.31 (C-3) and the correlation of *δ*_H_ 4.55 (H-1 of Xyl) with *δ*_C_ 82.25 (C-2″) were observed, which indicated that the sequence of the saccharide chain of C-3 was xylosyl-(1→2)-glucopyranosyl-(1→3). QSB was also isolated from EUP for the first time.

**Compound 4**: light yellow powder. ESI-MS (*m*/*z*): 611.1620 [M + H]^+^ (positive), 609.1487 [M − H]^−^ (negative), C_27_H_30_O_16_ (Cal: 610.1534). Compound **4** was identified as quercetin 3-*O*-neohesperidoside (QNH) by comparison with previously reported spectral data [40,41]. The β-configuration of the glucopyranosyl group was indicated based on the large coupling constants (*J*_1,3_ > 7.0 Hz) of the anomeric protons. In the HMBC spectrum (Figures S3 and S6), the correlation of *δ*_H_ 5.64 (H-1 of 3-*O*-Glc) with *δ*_C_ 133.30 (C-3) and *δ*_C_ 77.74 (C-3″) and the correlation of *δ*_H_ 5.07 (H-1, Rha) with *δ*_C_ 77.84 (C-2″) were observed, which indicated that the sequence of the saccharide chain of C-3 was rhamnopyranosyl-(1→2)-glucopyranosyl-(1→3). QNH was isolated from EU for the first time.

In addition, the results of the HPLC peak-area normalization showed that the purities of the three compounds were above 95%.

### 2.4. Results of the Molecular Docking

**Figure 5:**
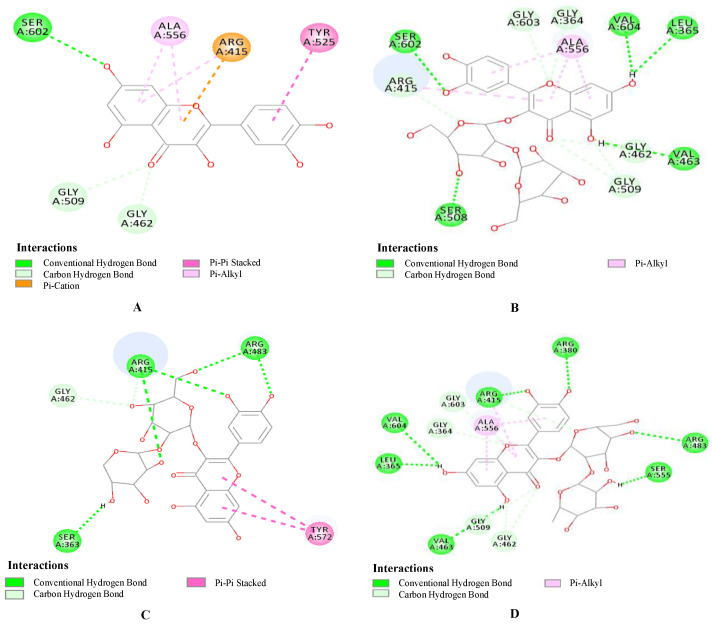
Interaction of (**A**) quercetin (control), (**B**) QSH, (**C**) QSB, and (**D**) QNH with KEAP1 receptors.

The interaction energy states a total for all of the types of interactions, such as the van der Waals force, hydrogen bonding, the charge effect, hydrophobic interactions, etc. Figure 5 exhibits the 2D interaction diagrams for the separated compounds. As displayed, all three compounds had higher binding affinity values than quercetin. QSH exhibited the highest interaction energy of 71.67 kcal/mol with the carbon–hydrogen bonds of Ser602, Val604, Leu365, Val463, Ser503, Arg415, Gly462, Gly509, Gly603, and Gly364, followed by QNH, which showed an interaction energy of 61.62 kcal/mol with the carbon–hydrogen bonds of Arg380, Arg415, Gly603, Gly364, Val604, Leu365, Val463, and Gly509, while QSB showed the lowest interaction energy of 54.67 kcal/mol with the carbon–hydrogen bonds of Arg415, Arg483, Gly462, and Ser363. The interaction energy of quercetin is only 34.0138.

### 2.5. Cytotoxicity Assay and the Antioxidant Effect

**Figure 6:**
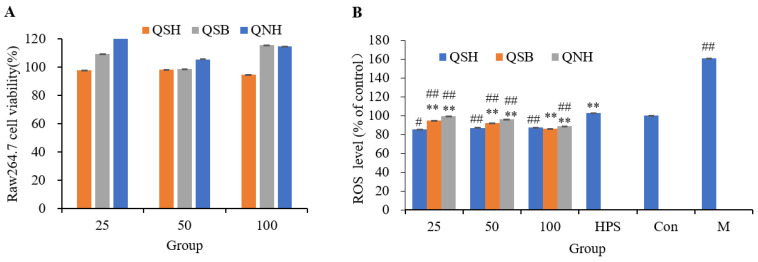
Cell experiment results. (**A**) Effects of different concentrations of QSH, QSB, and QNH on the activity of Raw264.7 cells, (**B**) Effects of different concentrations of QSH, QSB, and QNH on ROS production in Raw264.7 cells. DCF fluorescence was quantified in control (Con) and Raw264.7 cells incubated in the absence (M) or in the presence of QSH, QSB, or QNH for 24 h. Data are expressed as a percentage of control. Values are the mean ± SD from three independent experiments. (HPS) quercetin, (M) Model. Significant differences are denoted by symbols: ## *p* < 0.01, # *p* < 0.05 vs. control; ** *p* < 0.01 vs. M.

Three different concentrations (25, 50, and 100 μM) were studied in our experiments, and the results are summarized in Figure 6A. As shown, all compounds exhibited cell viability at the tested concentrations. The results demonstrated that the maximum concentration of 100 μM could be used for subsequent antioxidant experiments. A concentration-dependent study of viability losses was investigated in RAW264.7 cells induced by H_2_O_2_. After treatment with increasing concentrations of H_2_O_2_ for 4 h, the cell viability was determined using the CCK 8 method. As shown in Figure S7, gradual reductions in cell viability were found with increasing concentrations of H_2_O_2_. Based on the results, RAW264.7 cells were treated with 1.0 mM H_2_O_2_ for 4.0 h, and the cell viability was about 50.52 ± 1.48%. Finally, we chose a concentration of 1.0 mM for further experiments.

The effects of QSH, QSB, and QNH on the intracellular ROS levels of RAW264.7 cells are shown in Figure 6B. Treatment with 1.0 mM H_2_O_2_ significantly increased the intracellular ROS levels. As indicated, all tested compounds exhibited significant protective effects against H_2_O_2_-induced oxidation damage, even at the lowest concentration, compared to the positive control (quercetin). This was consistent with the results of the molecular docking.

### 2.6. Development and Validation of the Quality Standard

#### 2.6.1. Optimization of the Extraction

The extraction solvent, solid–liquid ratio, and extraction time have important effects on the extraction of target constituents in EUP. In order to obtain the proper extraction efficiency of QSH, QSB, and QNH, single-factor tests were performed for the extraction time (15, 30, 45, and 60 min), the extraction solvent (methanol, 50% methanol, ethanol, and 50% ethanol (*v*/*v*)), and the solid–liquid ratio (1:50 g·mL^−1^, 1:100 g·mL^−1^, and 1:250 g·mL^−1^). Finally, by comparing the extraction yields of the three constituents in a 50% methanol solvent, the best extraction method for UHPLC-QTOF-MS was 0.5 g of the sample powder extracted with 50% methanol (25 mL) on an ultrasonic machine for 30 min.

#### 2.6.2. Optimization of Chromatographic Conditions

To establish an efficient and accurate content determination method, the chromatographic column type, mobile phase composition, and current speed were optimized. Then, we found that when the Halo Phenyl-Hexyl column was selected, and acetonitrile–0.2% phosphoric acid solution was used as the mobile phase, with a flow rate of 0.5 mL·min^−1^, the Q-markers could be resolved well, the symmetry and shape of the peaks were good, and the elution time was short.

#### 2.6.3. Validation of the Analytical Method

We developed a simultaneous HPLC analysis method using the three markers as indicators for the efficient quality control of EUP. The assay was tested with several parameters, including linearity, stability, precision, repeatability, and recovery. The coefficient of determination (r^2^), which evaluates linearity, showed excellent linearity from 0.9999 to 1.0000 for all markers based on the prepared calibration curve (QSH, y = 665.43x + 0.18; QSB, y = 591.50x + 0.77; QNH, y = 666.92x + 0.60), and the linear ranges were 0.0128 to 1.27 mg·mL^−1^, 0.0157 to 0.786 mg·mL^−1^, and 0.0117 to 0.392 mg·mL^−1^, respectively. All RSD values of the repeatability, precision, and stability of the investigated markers were <1.55%. The recoveries (%) of compounds **1**–**3** ranged from 98% to 102% for each concentration level, and the RSDs were less than 2%. These results demonstrated that the sensitivity and applicability of the optimized HPLC-PDA were feasible for the quantitation analysis of the three Q-markers in pollen.

#### 2.6.4. Sample Analysis

The contents of the three Q-markers from the EUP samples of different batches are given in Table 4. The results showed that the contents of the Q-markers in S33 and S34 were 0; that is, they did not contain these three components, which proved that these two batches of samples were fake, and the microscopic identification results also proved this. Samples from other batches contained Q-markers, and the content distribution ranges of QSH, QSB, and QNH were 9.12 to 14.74 mg/g, 7.29 to 10.52 mg/g, and 2.05 to 4.05 mg/g, respectively. This shows that this method can scientifically and accurately identify the authenticity of EUP, and it is necessary to establish a quality evaluation method for EUP.

**Figure 7:**
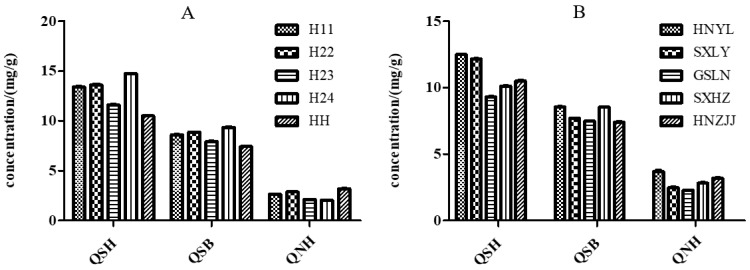
The influence of variety and producing areas on the quality of EUP. (**A**) The influence of variety on the quality of EUP. (**B**) The influence of producing areas on the quality of EUP.

Four varieties and mixed pollen were tested, and it was found that the contents of the three quality markers in the EUP of different varieties were within the normal ranges (Figure 7A). In addition, pollen from different producing areas was compared, and there were no significant differences in the contents of quality markers in pollen from five places (see Figure 7B).

## 3. Discussion

EU has a long history of application as a traditional Chinese medicine in China. So far, more than 200 compounds have been isolated and identified from EU. There are many articles on the chemical composition and quality evaluation of *Eucommia ulmoides* leaves, bark, and male flowers. EUP, as a non-medicinal part of EU that is rich in nutrients, trace elements, and minerals [28], has been increasingly studied in recent years. However, the quality control of EUP has not been well established due to the lack of quality markers (Q-markers). The chemical components in EUP were identified using UPLC-QTOF-MS and compared with their relative peak areas in EUB, EUL, and EUF. The results suggested that flavonoids are its characteristic components.

Oxidative stress has been shown to participate in a wide range of diseases, including cardiovascular disease [42], Alzheimer’s disease [43], male infertility [44,45], and cancer [46]. The application of antioxidants can alleviate oxidative stress-induced disease progression. Considering safety, the discovery of natural antioxidants has received increasing attention in recent years. As a new functional food ingredient, EUP has significant antioxidant activity, which may be related to its high content of polyphenolic compounds. Despite the employment of many methods in the extraction of antioxidants from EU, the traditional strategy is time-consuming, cumbersome, and less efficient for screening. The DPPH-HPLC active component detection method is convenient, fast, and highly accurate, and it is widely used for screening antioxidant components in plants [47,48,49]. After interaction with DPPH, the UV absorption of free radical-scavenging compounds decreased or disappeared, and identity confirmation could be achieved using the UPLC–DAD–TOF/MS technique.

Using this method, five components with antioxidant activity in EUP were selected, all of which had phenolic hydroxyl structures, which were considered to be the main reason for their DPPH radical-scavenging ability. QSH, QSB, and QNH had larger UV absorption at 254 nm before the DPPH reaction, which significantly decreased or even disappeared after the reaction, indicating that these three compounds showed higher antioxidant capacities compared to other components [39].

Kelch-like ECH-associated protein 1 (Keap1), an adaptor of the E3 ligase complex that promotes the degradation of nuclear factor erythroid 2-related factor 2 (Nrf2), is a master transcriptional regulator in the antioxidative response. The KEAP1–Nrf2 signaling pathway senses reactive oxygen species and regulates cellular oxidative stress. Inhibiting KEAP1 to activate the Nrf2 antioxidant response has been proposed as a promising strategy to treat chronic diseases caused by oxidative stress [50,51,52]. The higher the binding energy with KEAP1, the better the antioxidant capacity of the compound.

Three flavonoids were successively isolated and purified from a 50% methanol extract of EUP for the first time under the guidance of the DPPH-HPLC method. The molecular docking results and in vitro cell experiments both indicated that QSH, QSB, and QNH have significant antioxidant activity, which is the same as the results reported in the literature [37]. Semi-quantitative results showed that the peak-area ratio of the three compounds in pollen was significantly higher than that in other organs. Considering that they are also characteristic components of EUP, selecting these three components as indicators to evaluate the quality of EUP is considered scientific and reasonable. EU has many varieties, such as Huazhong 11, Huazhong 22, Huazhong 23, Huazhong 24, etc. Different varieties and environments may affect the composition of bioactive chemicals in EUP. This study only explored the two key factors that may affect the quality of pollen, namely, origin and variety, but not other factors that may affect the quality of pollen, such as the harvest time and original processing methods, which will continue to be studied in the future.

## 4. Materials and Methods

### 4.1. Materials and Apparatus

The EUP was collected from Xuchang, Henan province; Hanzhong, Shanxi province; Longnan, Gansu province; and Zhangjiajie, Hunan province, China. The EUL, EUF, and EUB samples were collected from Xuchang Henan province. Samples S1–S34 were authenticated by Professor Zhimin Wang at the Institute of Chinese Materia Medica, China Academy of Chinese Medical Sciences, Beijing, P.R. China. Chemical reference substances (CRSs), including aucubin, geniposidic acid, chlorogenic acid, asperuloside, quercetin-3-*O*-sophoroside, quercetin-3-*O*-sambubioside, quercetin-3-*O*-neohesperidoside, rutin, quercetin, asperuloside, hyperoside, genipin acid, chlorogenic acid, caffeic acid, gallic acid, luteolin, oleanolic acid, neochlorogenic acid, cryptogenic acid, naringin, naringenin, scutellarin, ursolic acid, isochlorogenic acid A, isochlorogenic acid B, isochlorogenic acid C, and genipin, were purchased from Herb Purify Biological Technology Co., Ltd., Chengdu, China. The purity of all CRSs was over 98%. DPPH was purchased from Coolaber Science & Technology Co., Ltd., Beijing, China. Acetonitrile and methanol were purchased from Fisher, Waltham, MA, USA, and were chromatographically pure. The water was distilled water, and other analysis-grade reagents were purchased from Sino Pharm Chemical Reagent Co., Shanghai, China. Fetal bovine serum was purchased from Beijing Pulilai Gene Technology Co., Ltd. (Beijing, China). Streptomycin, phosphate-buffered saline, Dulbecco’s modified Eagle medium (DMEM), and penicillin were purchased from Beijing Dongdu Kaiyuan Biotechnology Co., Ltd. (Beijing, China). The RAW264.7 cell line was purchased from the Chinese Type Culture Collection. An HC-2518 high-speed centrifuge (Anhui ustic zonka scientific instruments Co., Ltd., Hefei, China), a Xevo g2-s QTOF mass spectrometer, a Waters ACQUZTY UPLC system (Waters Technologies, Shanghai, China), an HC-2518 fragmentation voltage (Anhui Zhongke Zhongjia Science Co., Ltd., Hefei, China), a KQ-250DE CNC Ultrasonic Cleaner (Kun Shan Ultrasonic Instruments Co., Ltd., Kunshan, China), an Ultimate3000 high-performance liquid chromatography system (ThermoFisher Co., Ltd., Waltham, MA, USA), an LC3000 preparative HPLC system (Beijing Chuangxintongheng Science & Technology Co., Ltd., Beijing, China), and a Halo Phenyl-Hexyl column (4.6 × 150 mm, 2.7 μm; Waters Technologies, Milfordcity, MA, USA) were used.

### 4.2. Preparation of Sample

About 0.5 g of the sample powder was precisely weighed. Then, 25 mL of 70% methanol was precisely added, weighed, extracted via sonication (250 W, 40 kHz) for 30 min at room temperature, and cooled. The weight loss comprised 70% methanol, and the solution was shaken well and filtered. The filtrate was centrifuged at 12,000 r·min^−1^ for 10 min, and the supernatant was filtered through a 0.22 µm-microporous membrane and stored at 4 °C in a refrigerator.

### 4.3. UPLC-ESI-TOF/MS Analysis

The extracts of EUP were analyzed using UPLC-ESI-TOF/MS, which consisted of a Waters ACQUZTY UPLC system (Waters Technologies, Milford, MA, USA) coupled to a Xevo G2-S QT mass spectrometer (Waters Technologies, USA). An ACQUITY UPLC^®^ BEH C_18_ column (1.7 μm, 2.1 × 100 mm, Waters Technologies, USA) was used during the analysis, and the temperature of the column was maintained at 40 °C. The flow rate was 0.3 mL/min, the injection volume was 2 μL, and the determination wavelength was set at 190–400 nm. The mobile phases were composed of A (water containing 0.1% acetic acid (*v*/*v*)) and B (acetonitrile). The linear gradient program was as follows: 0–5 min, 98% A; 5–35 min, 98–5% A; 35–40 min, 5% A. Mass spectrometry was carried out in the scan mode from 50 *m*/*z* to 1200 *m*/*z* using both negative and positive modes at 450 °C with a corona discharge at ±6.0 kV. The ESI-MS conditions were as follows: the capillary voltage was set to 2.0 kV; the temperature was 120 °C; the drying gas flow was 10.0 L/min; and the nebulizing gas pressure was 45 psi.

### 4.4. Screening Active Compounds by HPLC-DPPH

First, an HPLC analysis of eight standards—geniposidic acid, chlorogenic acid, asperuloside, geniposide, QSH, QSB, QNH, and rutin—was optimized to obtain a baseline separation. This analysis was carried out using a U3000 system equipped with a diode array detector (PDA) system, a column oven, and an automatic injector. The injection volume was 10 μL. A Halo Phenyl-Hexyl column (2.7 μm, 4.6 × 150 mm) was used during the analysis, and the temperature of the column was 30 °C. The flow rate was 0.5 mL/min, the injection volume was 10 μL, and the determination wavelength was set at 254 nm. The mobile phases were composed of A (acetonitrile) and B (water containing 0.2% phosphoric acid (*v*/*v*)). The linear gradient program was as follows: 0–2 min, 1% A; 8–18 min, 1–7% A; 18–60 min, 7–17% A. There was a 10-min-post run to re-equilibrate the column for each run. First, the sample solution was analyzed by HPLC to obtain as much chemical information as possible. A DPPH-radical solution was freshly prepared in methanol. The EUP extract was mixed with a DPPH-methanol solution (2 mg/mL) at a ratio of 1:1 (*v*/*v*). After incubation in the dark at 25 °C for 60 min, the mixture was centrifuged at 12,000 rpm for 10 min, and then the supernatant was analyzed by the same chromatographic condition. By comparing the chromatographic profiles of DPPH-reacted samples and control samples, the main antioxidants in the EUP extract could be screened.

The EUP extract was analyzed by HPLC–ESI-Q-TOF-MS. The chromatographic conditions were the same as those in Section 4.4, except for replacing phosphoric acid with formic acid. The mass spectrometry conditions were the same as those in Section 4.3.

### 4.5. Preparation and Characterization of the Main Active Components

#### 4.5.1. Analytical Condition

An LC3000-prep-HPLC system was used to accomplish the preparation and characterization of the active components screened from EUP. A Kromasil 100-5 C_18_ column (250 × 100 mm, 10 mm; Waters Technologies, USA) was used; the flow rate was maintained at 2.0 mL/min; the injection volume was 50 mL; the determination wavelengths were set at 254 nm and 353 nm; and the mobile phases were composed of A (methanol) and B (water containing 0.2% acetic acid (*v*/*v*)). The linear gradient program was as follows: 0~10 min, 20→36% A; 10~32 min, 36% A; 32~33 min, 36→80% A; 33~41 min, 80% A; 41~41.5 min, 80→20% A; 41.5~48 min, 20% A. For the GC analysis, an Optima 5MS capillary chromatographic column (320 μm × 0.25 μm, 30 m, MACHEREY-NAGEL) and an FID detector were used. The temperature program was as follows: an initial temperature of 170 °C for 3 min; a temperature increase of 2 °C/min to 230 °C for 5 min; carrier gas: N_2_; injection temperature: 250 °C; detector temperature: 300 °C; nitrogen gas flow: 1 mL/min; hydrogen gas flow: 30 mL/min; airflow: 50 mL/min; injection volume: 2.0 μL; injection method: split injection; split ratio: 60:1.

#### 4.5.2. The Derivatization Procedures of Quercetin Di-Glycoside

The pretreatment of samples and the derivatization of monosaccharides were carried out according to previous studies [53,54].

#### 4.5.3. Nuclear Magnetic Resonance Spectroscopy (NMR)

The sample was dissolved in DMSO-*d*_6_ (0.5 mL). The ^13^C-NMR, ^1^H-NMR, COSY, TOCSY, DEPT, HMQC, and HMBC spectra were recorded at 298 K using a JNM-ECP 600 MHz NMR spectrometer.

### 4.6. Molecular Docking

To confirm the interactions between the core antioxidant targets and the components, molecular docking was conducted by selecting the key oxidative stress protein KEAP1, with a high median degree value, as a receptor and the three isolated compounds as the ligands. The non-mutated tertiary structure of the targeted protein (KEAP1, PDB code: 4XMB) was initially downloaded in the PDB format from the Protein Data Bank (https://www.rcsb.org/, accessed on 26 September 2022). The 3D chemical structures of the candidate compounds were drawn and saved in the SDF format. All the documents were converted to the PDB format for subsequent molecular docking. In the Discovery Studio 2020 software, the water molecules were deleted from the ligands, nonpolar hydrogen was added, and the Gasteiger charge was calculated. The potential core ligands were subjected to the energy minimization treatment, and the ligand atom type was obtained after a calculation. The Discovery Studio 2020 software was used for the calculation of the docking of semisoft molecules.

### 4.7. Antioxidant Activity Evaluation of Each Compound

#### 4.7.1. Cell Culture and Cell Viability Assay

The RAW264.7 cell line was purchased from the Chinese Type Culture Collection (NICR, Beijing, China). The cell lines were grown in DMEM with 10% FBS and 1% P/S and were incubated at 37 °C in 5% CO_2_. To determine cell viability, RAW264.7 cells were seeded into a 96-well plate at a density of 1 × 10^4^ cells/well, followed by treatment with compounds at 25, 50, and 100 μM. The results were expressed as the mean cell survival, normalized to the control, as determined using a CCK-8 assay, according to the manufacturer’s protocols.

#### 4.7.2. Detection of Intracellular ROS Generation

Intracellular ROS levels were measured using the fluorescent probe DCFH-DA, which, after crossing the plasma membrane, is hydrolyzed to DCFH and oxidized to the fluorescent product DCF. Sample stock solutions were prepared in DMSO. RAW264.7 cells were grown in 96-well plates at 1 × 10^4^ cells/well and cultured for 24 h at 37 °C with 5% CO_2_. RAW264.7 cells were incubated with a DMEM culture. The sample solutions were added to the cell culture for 24 h. Each treatment was exposed to a medium containing H_2_O_2_ (1.0 mM) for another 4 h, while the medium in the control group was only replaced with a complete medium for another 4 h. Next, the treated RAW264.7 cells (1 × 10^4^/well in 96-well plates) were incubated in a cell medium containing 5 μM DCFH-DA for 30 min, followed by three washes using a serum-free cell-culture medium. The fluorescence intensity was measured using a Varioskan Flash full-wavelength multifunctional microplate reader with excitation at 485 nm and emission at 530 nm.

### 4.8. Development and Validation of the Quality Standard

#### 4.8.1. Chromatographic Conditions

The extracts of EUP were analyzed using UHPLC. A Halo Phenyl-Hexyl column (2.7 μm, 4.6 × 150 mm) was used during the analysis, and the temperature of the column was set at 30 °C. The flow rate was 0.5 mL·min^−1^, the injection volume was 10 μL, and the determination wavelength was set to 254 nm. The mobile phases were composed of A (acetonitrile) and B (water containing 0.2% phosphoric acid (*v*/*v*)). The linear gradient program was as follows: 0–2 min, 1% A; 8–18 min, 1–7% A; 18–60 min, 7–17% A.

#### 4.8.2. Validation of the Method

The quantification method of the three Q-markers was validated with respect to linearity, stability, precision, repeatability, and recovery in accordance with the guidelines for the validation of analytical methods of the Chinese Pharmacopoeia (fourth part) (Chinese Pharmacopoeia, 2020). Serial dilutions of mixed standards were used to establish the standard curves, and the linear regression equation correlation coefficient and linear range were calculated. For precision, the solutions were examined in triplicate for 3 consecutive days. To validate the repeatability, six samples of EUP were accurately weighed and prepared independently, according to the optimal conditions above, and then analyzed. The same sample solution was taken and determined at 0, 2, 4, 8, 12, 24, and 48 h after its fresh preparation, according to the above chromatographic conditions, to evaluate the stability. Recovery experiments were used to assess the accuracy of the method. Standards at three different concentration levels, including low (80%), median (100%), and high (120%) levels, were added to samples with known content. Each experiment was repeated three times, and the spiked samples were analyzed using UHPLC-PDA to evaluate the recoveries. The recoveries were calculated using the following formula: recovery (%) = (detected amount − original amount)/spiked amount × 100%.

### 4.9. Statistical Analysis

The obtained data were analyzed with SPSS (version 21) software using a one-way ANOVA, followed by an LSD post hoc test. The data were represented as means ± SDs, and *p* < 0.05 indicated statistical significance.

## 5. Conclusions

The chemical composition of EUP was investigated for the first time, providing a foundation for further in-depth research. Three characteristic active ingredients in EUP had strong and clear effects on DPPH while significantly reducing the production of ROS in RAW264.7 cells induced by H_2_O_2_. To evaluate and monitor the quality of EUP more scientifically, this study took the discovery and determination of Q-markers as the main finding and established a fast, sensitive, and characteristic evaluation method for the first time. We believe that this work can provide a new quality assessment model and a demonstration for the further development and utilization of EUP in the food or nutrition industries. This study only discussed two key factors that may affect pollen quality, namely, origin and variety, without exploring other factors that may affect pollen quality, such as the harvest time and original processing methods, which will continue to be studied in the future.

## Figures and Tables

**Table 1:** LC-MS analysis of chemical components in different organs of EU.

Peak Number	*t*_R_/(min)	Molecular Weight	[M + H]^+^/[M + Na]^+^/[M + NH_4_]^+^ (Error, ppm)	[M − H]^−^/[M + HCOO]^−^ (Error, ppm)	MS/MS Fragments (P)	MS/MS Fragments (N)	Molecular Formula	Compound	Part
**1**	0.85	342.1162	365.1049 (−1.4)	387.1140 (0.35)	163.0633, 119.0341, 225.0872, 164.0695	191.0561, 195.0515, 129.0197, 101.0244	C_12_H_22_O_11_	Isomaltose	EUB, EUL, EUF, EUP
**2**	0.91	164.0473	182.0802 (−2.7)		145.0482, 131.0497, 119.0479, 149.0592		C_9_H_8_O_3_	*p*-coumaric acid	EUL, EUF, EUP
**3**	2.61	350.1577	373.1462 (−1.9)	395.1553 (−0.1)	135.0785, 153.0897	195.0287, 153.0540, 149.0614	C_15_H_26_O_9_	Eucommiol II	EUB, EUL
**4**	2.80	350.1577	373.146 (−2.4)	395.1556 (0.7)	135.0785, 153.0897	195.0287, 153.0540, 149.0614	C_15_H_26_O_9_	Eucommiol II isomer	EUB, EUL
**5**	3.44	346.1264	369.1149 (3.3)		311.1122, 149.0590, 131.0484		C_15_H_22_O_9_	Aucubin *	EUL, EUF, EUP
**6**	3.44	392.1319		391.124 (−0.1)		295.1032, 345.1188, 227.0565, 183.0664	C_16_H_24_O_11_	Reptoside	EUB, EUL, EUF, EUP
**7**	3.74	390.1162	413.1052 (−1.9)	389.1078 (−1.5)	193.0480, 175.0373, 147.0429	371.0977, 227.0510, 165.0563	C_16_H_22_O_11_	Scandoside	EUB, EUL, EUF, EUP
**8**	4.07	154.0266	155.0339 (−3.4)	153.0192 (2.7)	137.0238	109.0312	C_7_H_6_O_4_	Protocatechuic acid	EUB, EUL, EUF, EUP
**9**	6.75	212.0685	213.0756 (−3.3)	211.0613 (3.09)	195.0647, 177.0538	149.0613, 193.0501	C_10_H_12_O_5_	C-veratroylglycol	EUB, EUL, EUF, EUP
**10**	6.98	374.1213	397.1092 (−4.7)	373.1132 (−0.7)	357.1173, 177.0544, 149.0593, 195.0649	211.0606, 193.0627, 167.0706, 149.0600, 123.0443	C_16_H_22_O_10_	Geniposidic acid *	EUB, EUL, EUF, EUP
**11**	6.99	404.1319		449.1295 (−0.0)	353.0863, 211.0611, 149.0608, 373.1136, 353.0863		C_17_H_24_O_11_	Deacetyl asperulosidic acid methyl ester	EUB, EUL, EUF, EUP
**12**	8.56	290.079	291.0855 (−4.7)		147.0428, 139.0374, 131.0479		C_15_H_14_O_6_	Catechin	EUL, EUF, EUP
**13**	8.72	432.1268	450.1606 (−1.22)	431.1179 (−2.4)	175.0388, 147.0435, 119.0485, 193.0522	373.1125, 257.1029, 211.0611, 251.0562	C_18_H_24_O_12_	Asperuloside acid *	EUL, EUF, EUP
**14**	8.77	354.0951		353.0854 (−2.4)		191.0565, 173.0457, 307.0816, 133.0295	C_16_H_18_O_9_	Chlorogenic acid *	EUB, EUL, EUF
**15**	8.79	332.1107	355.1018 (3.7)		181.0477, 179.0325		C_14_H_20_O_9_	Koaburaside	EUB, EUL, EUF, EUP
**16**	8.87	700.2579	718.2905 (−2.4)	745.2551 (1.2)	341.1376, 323.1269, 217.0853, 137.0584	699.2483, 583.1983, 537.1973, 375.1438, 341.1375, 359.1476, 195.0661, 137.0586	C_32_H_44_O_17_	Olivil 4,4″-di-*O*-b-d-glucopyranoside	EUB, EUF
**17**	8.93	180.0423	181.0499 (−1.0)	179.0344 (−0.2)	163.1225	135.0445, 161.0405, 117.0338	C_9_H_8_O_4_	Caffeic acid *	EUB, EUL, EUF, EUP
**18**	9.45	414.1162	437.1047 (−2.9)	459.1142 (−0.7)	175.0375, 163.0736, 131.0478	353.0867, 251.0597	C_18_H_22_O_11_	Asperuloside *	EUL, EUF, EUP
**19**	9.45	368.1107	391.0988 (−4.3)	413.1072 (−2.9)	353.0867, 147.0453		C_17_H_20_O_9_	Methyl chlorogenate	EUL, EUF, EUP
**20**	9.60	536.1894	537.1969 (−0.6)	535.1795 (0.7)	357.1323, 375.1438, 323.0546	373.1265, 343.1180, 285.1060, 520.1627	C_26_H_32_O_12_	(+)-1-Hydroxypinoresinol 4′-*O*-b-d-glucopyranoside	EUB, EUL
**21**	9.85	538.205	556.2395 (−0.2)	583.2031 (0.7)	341.1374, 345.1319, 137.0583, 311.0537	375.1444, 337.0927, 345.1324	C_26_H_34_O_12_	(−)-Olivil 4′-*O*-b-d-glucopyranoside	EUB, EUL, EUF, EUP
**22**	9.95	698.2422	699.2481 (−2.7)	743.2379 (−2.6)	519.1858, 375.1422, 327.1207	535.1737, 373.1261, 343.1265, 325.1095	C_32_H_42_O_17_	(+)-1-Hydroxypinoresinol 4′,4″-di-*O*-b-d-glucopyranoside	EUB
**23**	9.99	388.1369		433.1347 (−0.2)		375.1310, 207.0664, 175.0360, 371.0965	C_17_H_24_O_10_	Geniposide *	EUB, EUF, EUP
**24**	9.99	342.1315		387.1294 (−0.7)		165.0559, 123.0445, 147.0444	C_16_H_22_O_8_	(*E*)-Coniferin	EUB, EUF, EUP
**25**	9.99	180.0786	181.0862 (−1.5)	225.0765 (0.9)	149.0593, 163.0743, 131.0480	147.0460, 123.0454, 103.0151	C_10_H_12_O_3_	Pinusolidic acid	EUP, EUF
**26**	10.06	536.1894	537.1959 (−2.4)		375.1430, 357.1324, 519.1857		C_26_H_32_O_12_	(+)-1-Hydroxypinoresinol 4″-*O*-b-d-glucopyranoside	EUB
**27**	10.21	538.205	556.2386 (−1.4)	583.2023 (−0.6)	341.1373, 297.1113, 165.0677, 137.0585	375.1412, 277.1275, 507.1518, 123.0449	C_26_H_34_O_12_	(−)-Olivil 4″-*O*-b-d-glucopyranoside	EUB
**28**	10.31	342.1315		387.1288 (−0.8)		179.0518, 297.1067, 147.0453	C_16_H_22_O_8_	Coniferin	EUB, EUF, EUP
**29**	10.44	626.1483	627.1556 (−0.8)	625.1404 (−0.1)	465.1027, 303.0501, 285.0387, 247.0596, 153.0175	463.0869, 445.0765, 300.0279, 301.0325, 271.0241	C_27_H_30_O_17_	Baimaside *	EUL, EUF, EUP
**30**	10.55	682.2473	700.2802 (−2.1)	727.2450 (0.1)	357.1324, 235.0955	681.2398, 519.1866, 357.1339, 342.1103, 151.0398, 136.162	C_32_H_42_O_16_	(+)-Pinoresinol di-*O*-b-d-glucopyranoside *	EUB
**31**	10.55	682.2473	700.2796 (−2.9)	727.2444 (−0.7)	341.1380, 175.0742, 187.0725, 323.1268	519.1871, 357.1338, 327.1210	C_32_H_42_O_16_	(+)Dehydrodiconiferyl 4,γ-di-*O*-b-d-glucopyranoside	EUL, EUB, EUF
**32**	10.59	372.142		417.1399 (0.7)		179.0567, 162.0322	C_17_H_24_O_9_	Syringin	EUL, EUF, EUP
**33**	10.67	1086.996		1085.336 (0.2)		669.2391, 505.1715, 413.1081, 207.0661, 195.0657, 179.0563	C_48_H_62_O_28_	Ulmoidoside A	EUB, EUL
**34**	10.71	682.2473	700.2815 (−0.2)	727.2444 (−0.7)	311.1260, 323.0528, 571.1435	339.1228, 519.1867, 501.1761, 309.1124	C_32_H_42_O_16_	(+)Dehydrodiconiferyl 4,γ-di-*O*-b-d-glucopyranoside	EUL, EUB, EUF
**35**	10.8	712.2579	730.2915 (−1.0)	757.2547 (−1.1)	713.2574, 151.0378, 519.1867, 235.0946	491.1912, 545.1787, 387.1438, 372.1197	C_33_H_44_O_17_	(+)-Medioresinol di-*O*-β-d-glucopyranoside	EUB, EUL, EUF
**36**	10.83	226.0841	249.0732 (−2.8)	225.0767 (1.8)	209.0804, 163.0743, 149.0948, 227.0906	211.0611, 207.0662, 179.0350	C_11_H_14_O_5_	Genipin *	EUB, EUF
**37**	10.83	596.1378	597.1473 (2.7)	595.1305 (1.0)	303.0500, 153.0178, 465.1030	463.0869, 445.0771, 301.0322, 271.0247, 243.0295	C_26_H_28_O_16_	Quercetin 3-*O*-sambubioside *	EUL, EUF, EUP
**38**	10.89	568.2156	586.2477 (−3.9)	613.2128 (−0.7)	533.2036, 341.1376, 167.0690, 191.0697	405.1723, 537.2082, 371.1327, 531.1871, 207.0664	C_27_H_36_O_13_	Citrusin B	EUL, EUB, EUF
**39**	10.96	610.1534	611.1624 (−2.0)	609.1456 (0.1)	465.1025, 303.0501, 153.0177, 285.0384	463.0876, 445.0754, 301.0319, 151.0036	C_27_H_30_O_16_	Quercetin 3-*O*-neohesperidoside *	EUF, EUP
**40**	11.04	418.1628	419.1685 (−5.0)	417.1545 (−1.1)	401.1592, 371.1122	403.1431, 387.1094	C_22_H_26_O_8_	(+)-Syringaresinol	EUB, EUL
**41**	11.04	742.2684	760.3028 (0.0)	787.2652 (−1.1)	401.1591, 265.1058, 151.0375	579.2074, 417.1550, 551.1768, 403.1431, 387.1077	C_34_H_46_O_18_	Liriodendrin	EUL, EUB, EUF
**42**	11.27	610.1534	611.1618 (1.0)	609.1461 (0.9)	303.0496, 465.1016, 245.0456	301.0320, 271.0241, 255.0297, 243.0295, 227.0342, 151.0031	C_27_H_30_O_16_	Rutin *	EUL, EUF, EUP
**43**	11.45	258.0258	259.0599 (−2.9)	303.0508 (−1.1)	260.0669		C_14_H_10_O_5_	Alternariol	EUF, EUP, EUL
**44**	11.51	580.1792	581.1516 (1.7)	579.1355 (−0.9)	449.1081, 287.0601, 153.0118	463.0932, 284.0333, 255.0316, 227.0357	C_26_H_28_O_15_	Kaempherol-3-*O*-sambubioside	EUL, EUF, EUP
**45**	11.52	464.0955	465.1021 (−2.6)	463.0879 (0.5)		301.0335, 271.0299, 151.0038, 145.0291	C_21_H_20_O_12_	Isoquercitrin *	EUL, EUF, EUP
**46**	11.55	375.1438		421.1497 (−1.9)		360.1202, 227.0345, 271.0247, 345.1341	C_24_H_20_O_7_	(+) Cyclo-olivil	EUL, EUF, EUP
**47**	11.78	376.1522		375.1432 (−3.1)		225.0763, 308.1137, 327.1245, 357.1348, 343.1181	C_20_H_24_O_7_	(−)-olivil	EUB, EUL
**48**	11.79	550.205	568.2383 (−1.1)	595.2026 (−0.14)	435.1639, 329.1002, 321.1070	373.1267, 467.1566, 195.0661	C_27_H_34_O_12_	Eucommia A	EUL, EUB, EUF
**49**	11.88	908.3314	926.3583 (−2.9)	953.3279 (−1.8)	549.1991, 387.1425, 181.0482	745.2667, 583.2174, 387.1436, 195.0660	C_43_H_56_O_21_	Hedyotol C-4″,4‴-di-*O*-b-d-glucopyranoside	EUB, EUL, EUF
**50**	11.91	460.1006		505.0981 (−0.2)		445.0748, 443.0583, 177.0163, 145.0291, 151.0037	C_22_H_20_O_11_	Wogonoside	EUL, EUF, EUP
**51**	12.20	968.3525	986.3816 (−3.7)	1013.347 (−3.7)	775.2781, 549.1991, 417.1506, 417.1527	745.2667, 643.2385, 353.0870, 805.2880, 893.2989	C_45_H_60_O_23_	Guaiacylglycerol-b-syringaresinol ether-4″,4″-di-*O*-b-d-glucopyranoside	EUB, EUL
**52**	12.22	516.1628	517.1329 (−3.3)	515.1186 (−0.7)	499.1232, 287.0548, 135.0430	353.0876, 191.0557	C_25_H_24_O_12_	Isochlorogenic acid A *	EUB, EUL, EUF
**53**	12.24	448.1006		447.0927 (−0.1)		285.0390, 151.0032, 227.0347	C_21_H_20_O_11_	Astragalin *	EUL, EUF, EUP
**54**	12.34	520.1945	538.2272 (−3.0)	519.1864 (−0.5)	357.1323, 165.0685	357.1335, 342.1096, 136.0161	C_26_H_32_O_11_	(+)-Medioresinol di-*O*-b-d-glucopyranoside	EUB, EUL, EUF
**55**	12.54	580.2156	598.2487 (−2.1)	579.2074 (0.6)	417.1513, 247.0657	417.1551, 387.1456, 551.1286	C_28_H_36_O_13_	(−)-Syringaresinol-*O*-b-d-glucopyranoside	EUB, EUL, EUF
**56**	12.69	516.1628	517.1328 (−3.5)	515.1193 (−0.7)	499.1225, 163.0384, 179.0892, 135.0422	353.0886, 191.0561, 161.0237	C_25_H_24_O_12_	Isochlorogenic acid C *	EUB, EUL, EUF, EUP
**57**	12.81	188.1049	211.0942 (−2.0)	187.0973 (0.0)	135.0794, 153.0897, 107.0840	125.0973, 169.0862, 141.0919, 123.0813	C_9_H_16_O_4_	Eucommiol	EUB, EUF, EUP
**58**	13.16	374.1366	375.1433 (−2.9)	373.1286 (−0.3)	339.1217, 233.0795,	358.1068, 327.0871, 313.1084, 345.0982	C_20_H_22_O_7_	(+)-1-Hydroxypinoresinol	EUB, EUL
**59**	13.28	284.0685	302.1025 (0.6)	283.0606 (−2.1)	193.0478, 183.0309	147.0442, 136.0165, 125.0234	C_16_H_12_O_5_	Oroxylin A	EUF, EUP
**60**	13.55	196.1099	219.1001 (4.1)	241.1082 (2.5)	161.0595, 149.0576, 163.0358	163.0386, 145.0274	C_11_H_16_O_3_	Loliolide	EUB, EUL, EUF, EUP
**61**	14.32	302.0427	303.0498 (−2.2)	301.0345 (−1.1)	153.0171, 285.0373, 195.0272	151.0038, 285.0397, 271.0232	C_15_H_10_O_7_	Quercetin *	EUL, EUF, EUP
**62**	15.52	272.0685		271.0607 (−0.2)		151.0072, 119.0528, 93.0365, 177.0216, 227.0727	C_15_H_12_O_5_	Naringenin	EUL, EUF, EUP
**63**	15.53	270.0528	271.0597 (−3.5)	269.0453 (−1.1)	145.0630, 179.0329	177.0193, 145.0536	C_15_H_10_O_5_	Baicalein	EUL, EUF, EUP
**64**	15.76	286.0477	287.0558 (0.7)	285.0396 (−1.1)	153.0180, 179.0321	227.0337, 151.0026, 145.9311	C_15_H_10_O_6_	Kaempferol	EUL, EUF, EUP
**65**	21.27	172.1099		195.0999 (1.0)	95.0472, 121.0259		C_9_H_16_O_3_	1-Deoxyeucommiol	EUL, EUF, EUP
**66**	25.83	278.1518	301.1405 (−3.6)	277.1444 (1.5)	149.0217, 121.0270	121.0289	C_16_H_22_O_4_	1,2-benzenedicarboxylic acid bis(2-methylpropyl) ester	EUB, EUL, EUF, EUP
**67**	28.17	392.1471	410.1811 (−1.0)		313.0743, 185.0803		C_20_H_24_O_8_	Erythro-dihydroxydehydrodiconiferyl	EUB, EUL, EUF, EUP
**68**	28.17	184.0736	185.0810 (−2.1)		111.0070, 113.0218		C_9_H_12_O_4_	Eucommidiol	EUB, EUL, EUF, EUP
**69**	29.24	456.3604	457.3662 (−4.3)	455.3526 (0.2)	411.3613, 393.3506	277.2171, 407.1728, 377.1420	C_30_H_48_O_3_	Betulinic acid	EUB, EUL, EUF, EUP
**70**	29.32	456.3604	457.3699 (3.8)	455.3531 (−1.3)	439.3572, 393.3507, 411.3617, 203.1787	277.2171, 407.3311	C_30_H_48_O_3_	Ursolic acid *	EUL, EUF, EUP
**71**	30.83	256.2402	257.2474 (−2.6)		239.2364		C_16_H_32_O_2_	Palmitic acid	EUB, EUL, EUF, EUP
**72**	32.21	426.3862	427.3927 (3.0)		409.3821, 191.1783, 203.1777, 149.1315		C_30_H_50_O	Ulmoprenol	EUB, EUF, EUP
**73**	32.76	282.2559	283.263 (−2.5)	281.2484 (1.2)	265.2521, 137.1313, 123.1159	181.1241, 163.1133	C_18_H_34_O_2_	Oleic acid	EUB, EUL, EUF, EUP
**74**	36.14	576.439	599.4269 (−3.1)	621.4368 (0.3)	397.3818, 423.3231, 175.1460	473.2820, 283.1105	C_35_H_60_O_6_	Daucosterol	EUL, EUF, EUP

Note: * means determined by comparison with the reference sample; *t*_R_: retention time.

**Table 2:** Antioxidant components from EUP.

Peak Number	*t*_R_ (min)	Compound	Molecular Formula	Molecular Weight	[M + H]^+^/ [M + Na]^+^/[M + NH_4_]^+^ (Error, ppm)	[M − H]^−^/[M + HCOO]^−^ (Error, ppm)	MS/MS Fragments (P)	MS/MS Fragments (N)
**1**	20.72	Geniposidic acid *	C_16_H_22_O_10_	374.1213	397.1111 (0.1)	373.1131 (−1.0)	353.0553, 293.0344, 217.0472	211.0602, 149.0606, 123.0446
**2**	48.65	Quercetin 3-*O*-sophoroside *	C_27_H_30_O_17_	626.1483	627.1556 (−0.8)	625.1406 (0.2)	303.0506, 465.1033, 285.0383	271.0370, 301.0448, 463.0997
**3**	52.75	Quercetin 3-*O*-sambubioside *	C_26_H_28_O_16_	596.1378	597.1453 (−0.4)	595.1296 (−0.5)	303.0551, 465.1052, 285.0411	301.0448, 271.0336, 463.1041, 445.0971
**4**	53.62	Quercetin 3-*O*-neohesperidoside *	C_27_H_30_O_16_	610.1534	611.1602 (−1.7)	609.1453 (−0.4)	303.0504, 465.1023, 279.1604	300.0720, 301.0319, 271.0251, 445.0784
**5**	59.24	Isoquercitrin *	C_21_H_20_O_12_	464.0955		463.0876 (−0.1)		300.0259, 301.0337, 271.0238, 191.9362

Note: * means determined by comparison with the reference sample; *t*_R_: retention time.

**Table 3:** ^1^H (600 MHz) and ^13^C NMR (150 MHz) data of compounds **2**–**4** in DMSO-*d*_6_.

Position		Compound 2				Compound 3				Compound 4		
	*δ*_C_	*δ*_C_ Ref.	*δ*_H_ (*J* in Hz)	*δ*_H_ Ref.	*δ*_C_	*δ*_C_ Ref.	*δ*_H_ (*J* in Hz)	*δ*_H_ Ref.	*δ*_C_	*δ*_C_ Ref.	*δ*_H_ (*J* in Hz)	*δ*_H_ Ref.
**2**	156.66	157.07			155.61	156.70			156.52	156.32		
**3**	133.44	133.69			133.31	133.90			133.3	133.50		
**4**	177.88	178.35			177.63	178.30			177.71	177.58		
**5**	161.7	161.71	5-OH: 12.68 s		161.64	162.30			161.68	161.42	12.66 s	12.67 s
**6**	99.03	99.79	6.19 d	6.21 d	99.32	100.10	6.11 d	6.19 d	99.07	98.78	6.18 d	6.19 d
**7**	164.43	164.58	7-OH: 10.85 s		165.63	165.10			164.49	164.02	10.83 s	10.84 s
**8**	93.85	93.27	6.40 d	6.40 d	93.97	95.60	6.31 d	6.40 d	93.88	93.44	6.38 d	6.39 d
**9**	155.99	157.43			156.74	157.50			156.68	156.74		
**10**	104.37	103.59			103.92	104.90			104.43	103.87		
**1′**	121.57	121.63			121.5	122.10			121.62	121.96		
**2′**	115.81	114.74	7.55 d (2.2)	7.68 d	115.67	116.20	7.51 d (8.4)	7.56 d	115.53	115.84	7.52 d	7.53 d
**3′**	145.24	144.55	3′-OH: 9.71 s		145.41	149.50			145.27	144.95	9.72 s	9.17 s
**4′**	148.92	148.39	4′-OH: 9.21 s		149.14	151.10			148.79	148.18	9.16 s	9.73 s
**5′**	116.5	116.34	6.87 d (8.5)	6.90 d	116.33	119.30	6.80 d (8.4)	6.85 d	116.42	116.16	6.82 d	6.83 d
**6′**	122.25	121.63	7.61 dd (8.5, 2.2)	7.55 dd	122.27	122.60	7.62 d (8.4, 2.2)	7.66 d	122.09	121.15	7.59 dd	7.60 dd
**1″**	98.46	98.45	5.70 d (7.3)	5.37 d	98.39	95.50	5.68 d (6.7)	5.72 d	98.8	98.48	5.64 d (7.7)	5.65 d (7.6)
**2″**	83.14	81.49	3.46		82.25	81.70			77.91	76.76		
**3″**	76.97	74.15			76.52	77.50			77.74	77.34		
**4″**	70.07	69.53	3.03		70.04	69.40			70.7	69.86		
**5″**	77.18	76.50			77.26	76.10	3.43		77.84	77.15		
**6″**	61.17	60.91			61.01	60.50	3.51		61.39	60.52		
**1** **‴**	104.60	104.34	4.60 d (7.9)	4.77 d	104.92	104.40	4.55 d (7.3)	4.58 d	100.92	100.76	5.01 d	5.08
**2** **‴**	74.82	76.49	3.50		74.3	73.80			71.01	70.36		
**3** **‴**	76.97	76.85	3.44		76.52	76.70			71.07	70.83		
**4** **‴**	70.00	69.64			69.84	69.30			72.31	71.90		
**5** **‴**	77.93	76.66			66.02	65.60	3.65		68.67	68.57		
**6** **‴**	61.09	60.99							17.63	17.41	0.76	0.77

**Table 4:** The contents of Q-markers in thirty-four batches of EUP, as determined using the UHPLC/PDA method.

Number	Information	QSH (mg/g)	QSB (mg/g)	QNH (mg/g)
S1	HN2021	10.51	7.42	3.2
S2	20210323	11.26	7.76	3.76
S3	20210328	11.96	8.06	3.59
S4	20210331	13.53	8.84	3.21
S5	20210324	11.54	7.89	3.86
S6	20210325	13.88	8.94	3.00
S7	20210327	11.33	7.9	4.05
S8	20210404	13.25	8.77	3.49
S9	20210328	12.17	8.16	3.53
S10	20210410	13.62	8.84	3.64
S11	20210411	13.96	9.06	2.82
S12	20210413	11.42	7.85	3.08
S13	20210409	10.12	7.29	3.13
S14	Z20210330	13.44	8.77	3.17
S15	Z20210323	12.31	8.22	3.01
S16	Z20210325	12.95	8.58	2.88
S17	Z20210326	14.5	9.26	3.12
S18	Z20210331	13.42	8.75	3.28
S19	HNYY20210328	12.17	8.16	3.53
S20	HNYL202004	12.5	8.57	3.69
S21	SXLY202004	12.17	7.7	2.48
S22	GSLN202004	9.32	7.49	2.29
S23	SXHZ202004	10.13	8.55	2.81
S24	HNZJJ2022	10.43	7.84	2.86
S25	SXHZ2022	9.13	8.72	2.64
S26	20190430	12.67	7.77	3.80
S27	20190407	9.12	10.52	2.95
S28	20190404	9.95	9.13	2.66
S29	H11	13.4	8.61	2.64
S30	H22	13.67	8.87	2.88
S31	H23	11.61	7.93	2.12
S32	H24	14.74	9.36	2.05
S33 *	YHSW221207-1	0.00	0.00	0.00
S34 *	BZXH2022	0.00	0.00	0.00

Note: * The sample was fake.

## Data Availability

Not applicable.

## Supplementary Materials

The following supporting information can be downloaded at: https://www.mdpi.com/article/10.3390/molecules28135288/s1, Figure S1: UPLC/QTOF-MS based peak ion diagram (BPI) of different parts of EU.; Figure S2: Chromatogram for preparing liquid phase; Figure S3: Correlations and Key HMBCs of compounds **2**, **3** and **4**; Figure S4: ^1^H/^13^C HMBC spectrum of the compound **2** dissolved in DMSO-*d*_6_; Figure S5: ^1^H/^13^C HMBC spectrum of the compound **3** dissolved in DMSO-*d*_6_; Figure S6: ^1^H/^13^C HMBC spectrum of the compound **4** dissolved in DMSO-*d*_6_; Figure S7: Viability losses in RAW264.7 cells induced by various concentration of H_2_O_2_.

## Sample Availability

Samples of compounds **2**–**4** are available from the authors.

## Abbreviations

EU	*Eucommia ulmoides* Oliver
EUP	the pollen of *Eucommia ulmoides* Oliver
EUF	Male flowers of *Eucommia ulmoides* Oliver
DPPH	2,2-Diphenyl-1-picrylhydrazyl
HPLC	high-performance liquid chromatography
NMR	nuclear magnetic resonance
QSH	quercetin-3-*O*-sophoroside
QSB	quercetin-3-*O*-sambubioside
QNH	quercetin 3-*O*-neohesperidoside
NHC	the National Health Commission
