# Supplementary material for: Quality Marker Discovery and Quality Evaluation of Eucommia ulmoides Pollen Using UPLC-QTOF-MS Combined with a DPPH-HPLC Antioxidant Activity Screening Method
Source: Molecules. 2023 Jul 7;28(13):5288. doi: 10.3390/molecules28135288 (PMC10343934; doi:10.3390/molecules28135288)
Supplement: Supplementary file 1 [file molecules-28-05288-s001.zip › molecules-2418912-supplementary.pdf]

molecules

MDPI

Supplementary material

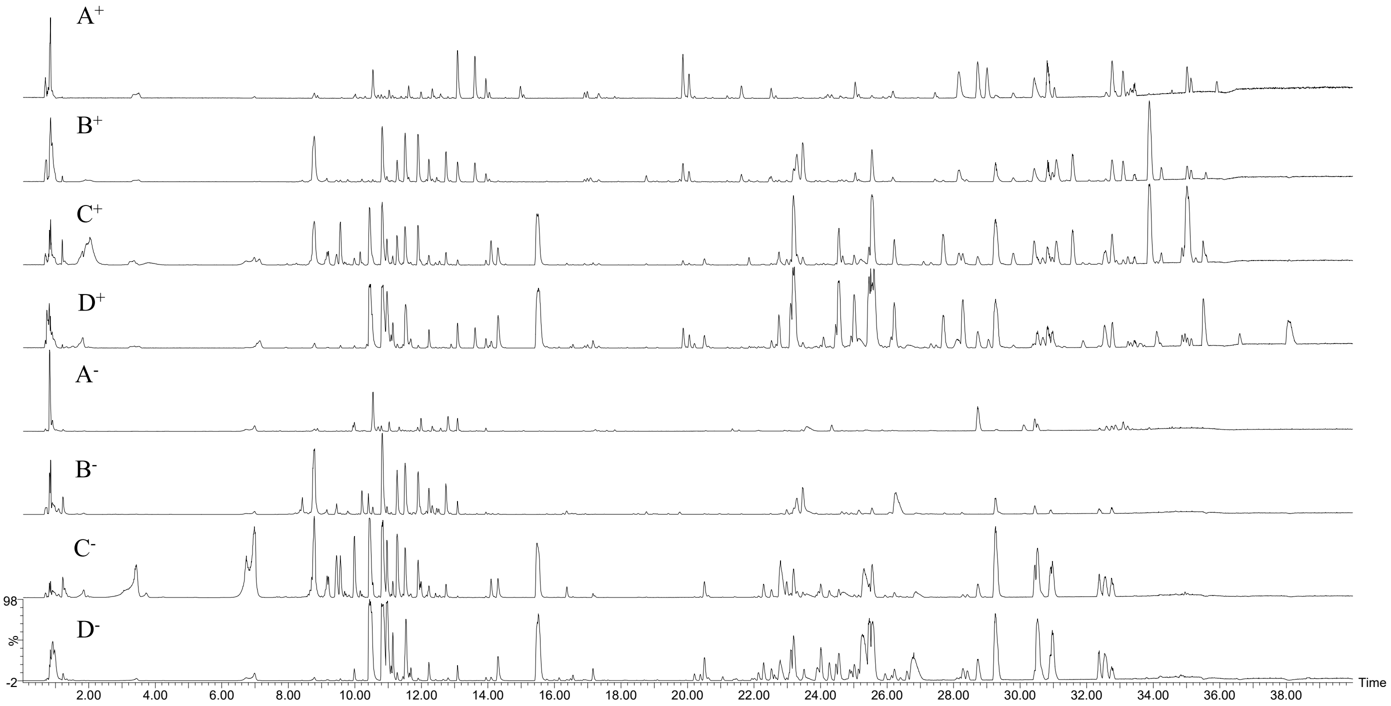

Figure S1 UPLC/QTOF-MS based peak ion diagram (BPI) of different parts of EU. A, EUB; B, EUL; C, EUF; D, EUP; +, positive; -, negative.

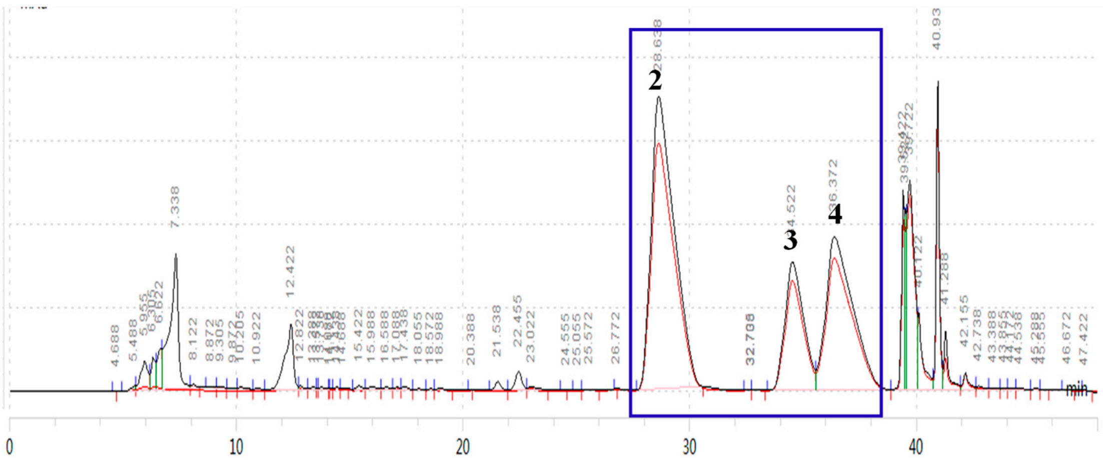

Figure S2 Chromatogram for preparing liquid phase.

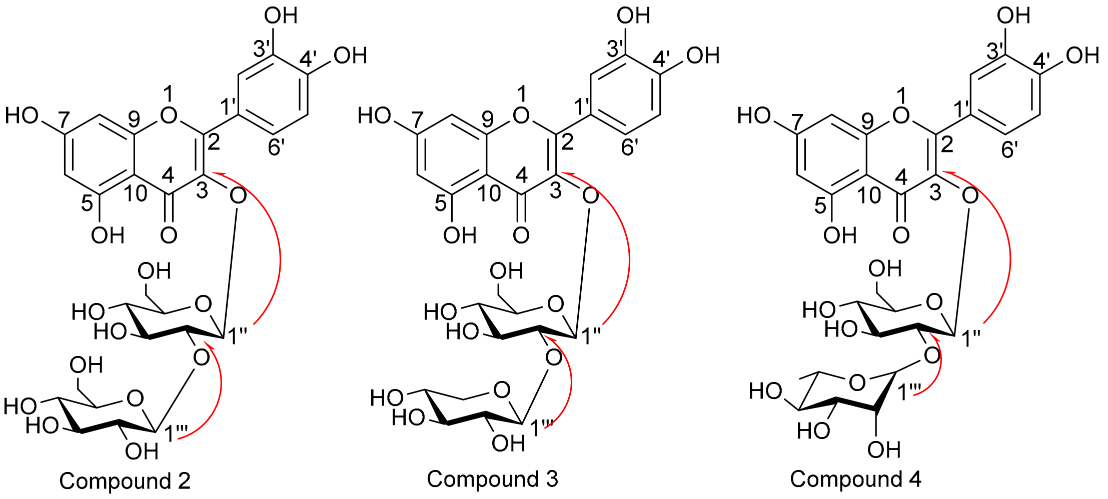

Figure S3 Correlations and Key HMBCs of compounds **2**, **3** and **4**. (HMBC correlation signals: H→C).

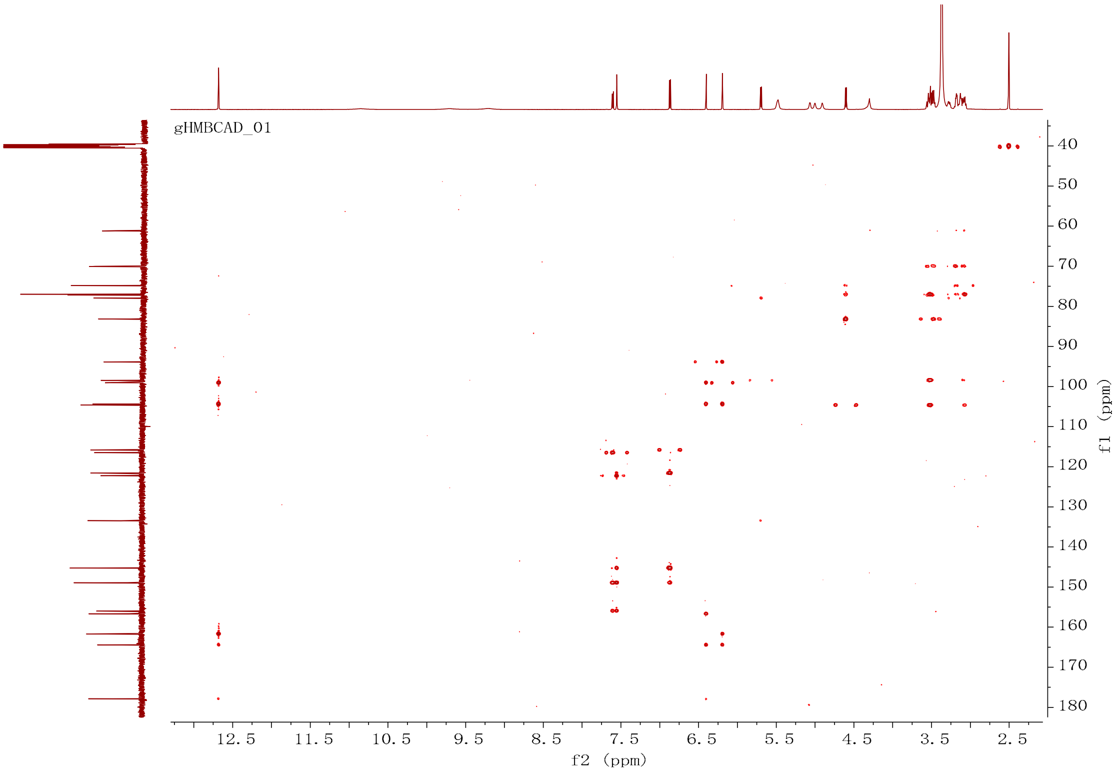

Figure S4 $^{1}H/^{13}C$ HMBC spectrum of the compound **2** dissolved in DMSO-d_6

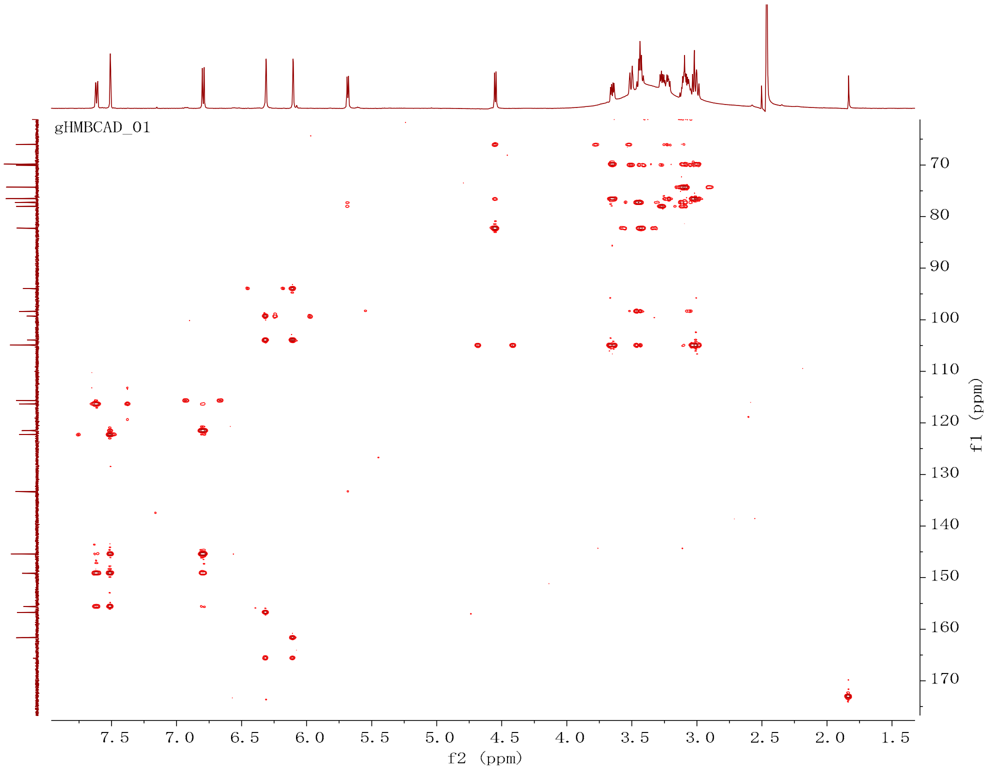

Figure S5 $^{1}H/^{13}C$ HMBC spectrum of the compound **3** dissolved in DMSO-d_6

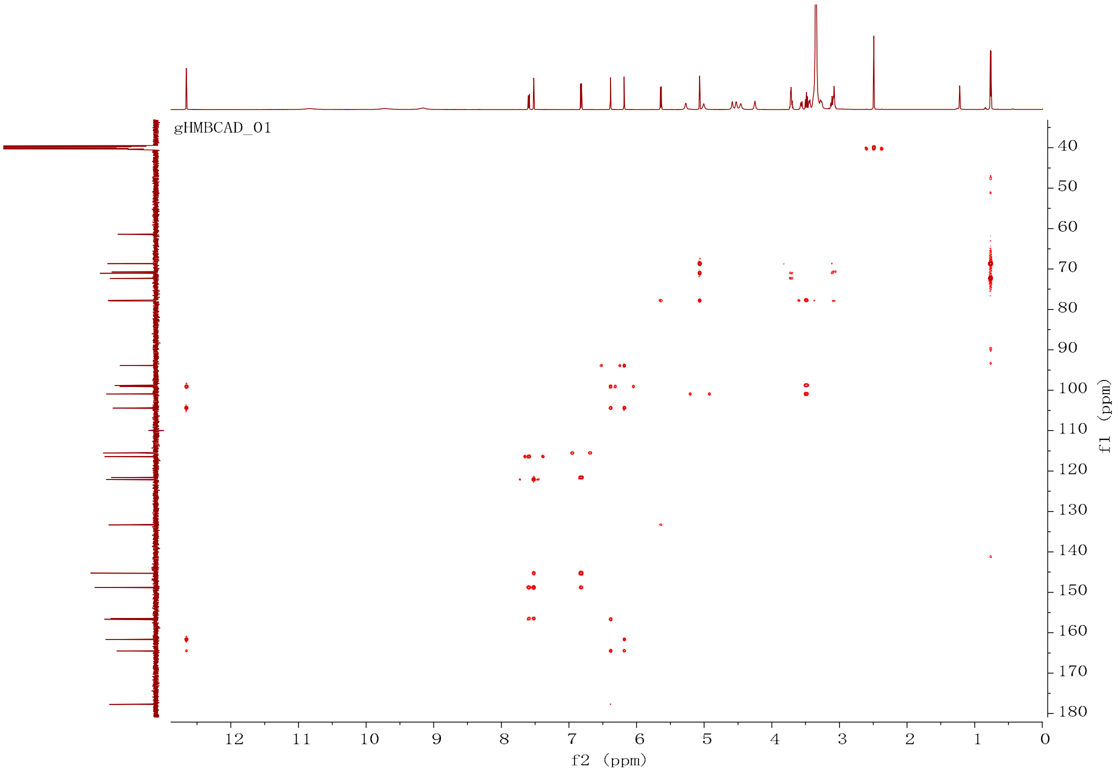

Figure S6 $^{1}H/^{13}C$ HMBC spectrum of the compound **4** dissolved in DMSO-d_6

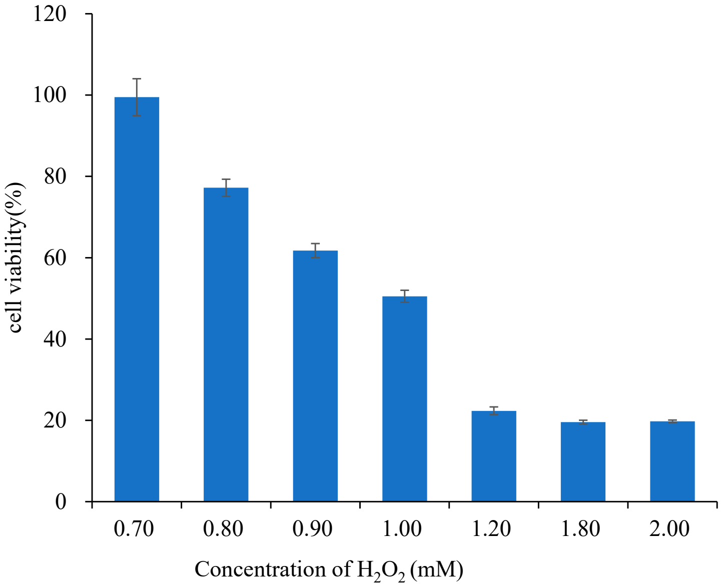

Figure S7 Viability losses in RAW264.7 cells induced by various concentration of H_2O_2
